# Supplementary material for: Bubbles enable volumetric negative compressibility in metastable elastocapillary systems
Source: Nat Commun. 2024 Jun 13;15:5076. doi: 10.1038/s41467-024-49136-w (PMC11176325; doi:10.1038/s41467-024-49136-w)
Supplement: Supplementary file 1 — Supplementary Information [file 41467_2024_49136_MOESM1_ESM.pdf]

SUPPLEMENTARY INFORMATION for Bubbles enable volumetric negative compressibility in metastable elastocapillary systems

Davide Caprini[1†], Francesco Battista[2†], Paweł Zajdel[3†], Giovanni Di Muccio[2†], Carlo Guardiani[2], Benjamin Trump[4], Marcus Carter[4], Andrey A. Yakovenko[5], Eder Amayuelas[6], Luis Bartolomé[6], Simone Meloni[7,*], Yaroslav Grosu[6,8,*], Carlo Massimo Casciola[2,*], Alberto Giacomello[2,*]

[1]Center for Life Nano- & Neuro-Science, Istituto Italiano di Tecnologia, Viale Regina Elena 291, 00161 Rome, Italy
[2]Dipartimento di Ingegneria Meccanica e Aerospaziale, Sapienza Università di Roma, Via Eudossiana 18, Rome, 00184, Italy
[3]A. Chełkowski Institute of Physics, University of Silesia, ul 75 Pułku Piechoty 1, Chorzów, 41-500, Poland
[4]Center for Neutron Research, National Institute of Standards and Technology, Gaithersburg, 20899, Maryland, USA
[5]X-Ray Science Division, Advanced Photon Source, Argonne National Laboratory, Argonne, 60439, Illinois, USA
[6]Centre for Cooperative Research on Alternative Energies (CIC energiGUNE), Basque Research and Technology Alliance (BRTA), Alava Technology Park, Albert Einstein 48, 01510 Vitoria-Gasteiz, Spain
[7]Dipartimento di Scienze Chimiche e Farmaceutiche, Università degli Studi di Ferrara, Via Luigi Borsari 46, I-44121, Ferrara, Italy
[8]Institute of Chemistry, University of Silesia, 40-006 Katowice, Poland

*To whom correspondence should be addressed: alberto.giacomello@uniroma1.it, ygrosu@cicenergigune.com, simone.meloni@unife.it, carlomassimo.casciola@uniroma1.it.

†These authors contributed equally to this work.

Contents:

Supplementary Text 1: Equilibrium solution for a MES. p. 2
Supplementary Text 2: Biological Pore Data. p. 3

Supplementary Figure 1: Experimental XRD pattern of the synthesised ZIF-67 sample. p. 4
Supplementary Figure 2: N_2 adsorption experiment in ZIF-67. p. 5
Supplementary Figure 3: Hydrophobic laminae experimental setup. p. 6
Supplementary Figure 4: Reversibility and repeatability of the milliMES cycles. p. 7
Supplementary Figure 5: MscL biological pore data. p. 8
Supplementary Figure 6: BK biological pore data. p. 9

Captions of Supplementary Movies 1 & 2. p. 10
References. p. 10

Supplementary Text 1

Equilibrium solutions for a MES

We consider an elastic solid of volume V, area A, and bulk modulus K under hydrostatic compression/decompression. For small volume changes, the total free energy variation with respect to the resting state reads:

$$\Delta\Omega = \frac{K}{2}\frac{(V-V_0)^2}{V_0} + \phi_{\mathrm{g}}(p-p_a)V + \alpha\gamma A\,, \tag{S1}$$

with V_0 the volume in the resting state, ϕ_{g} the volume fraction occupied by the gas, γ the liquid-vapour surface tension, and $\alpha = \phi_{\mathrm{lv}} + \phi_{\mathrm{sg}}\cos\theta_{\mathrm{Y}}$ as in the main text. Eq. (S1) can be linearised for small deviations $\delta a \equiv a - a_0$ from the resting lateral dimension a_0, using $V \approx V_0(1+3\delta a/a_0)$ and $A \approx A_0(1+2\delta a/a_0)$:

$$\Delta\Omega \approx \frac{9}{2}KV_0\frac{\delta a^2}{a_0^2} + \phi_{\mathrm{g}}(p-p_a)V_0\left(1+3\frac{\delta a}{a_0}\right) + \alpha\gamma A_0\left(1+2\frac{\delta a}{a_0}\right) \tag{S2}$$

Setting the first derivative of Eq. (S2) with respect to δa to zero yields:

$$\frac{\partial\Omega}{\partial\delta a} = 9KV_0\frac{\delta a}{a_0^2} + 3\frac{V_0}{a_0}\phi_{\mathrm{g}}(p-p_a) + 2\alpha\gamma\frac{A_0}{a_0} = 0 \tag{S3}$$

$$\Rightarrow \frac{\delta a}{a_0} = -\frac{\phi_{\mathrm{g}}(p-p_a)}{3K} - \frac{2}{9}\frac{\alpha\gamma}{K}\frac{A_0}{V_0}\ . \tag{S4}$$

At least two equilibrium configurations of the MES are possible, depending on the wetting state: the completely wet one corresponds to $\phi_{\mathrm{g}} = \alpha = 0$ and the dry one to $\phi_{\mathrm{g}} \approx 1$ (see main text):

$$V_{\mathrm{eq,wet}} = V_0 \tag{S5}$$

$$V_{\mathrm{eq,dry}} = V_0\left(1+3\frac{\delta a}{a_0}\right) = V_0\left(1-\frac{(p-p_a)}{K} - \frac{2}{3}\frac{\alpha\gamma}{K}\frac{A_0}{V_0}\right)\ . \tag{S6}$$

The linearised results above can be also seen as a monodimensional problem in which the elastic part is accounted for by a spring with constant $k = 9KV_0/a_0^2$. This leads to the simplified representation of the problem as two plates separated by a spring reported in the main text (Fig. 2), with the following results:

$$\frac{\delta a_{\mathrm{eq,dry}}}{a_0} = -\frac{3(p-p_a)}{k}\frac{V_0}{a_0^2} - \frac{2\alpha\gamma}{k}\frac{A_0}{a_0^2} \tag{S7}$$

$$\delta a_{\mathrm{eq,wet}} = 0\ . \tag{S8}$$

If one considers $V_0 = a_0^3$ and $A_0 = a_0^2$ the expressions above further simplifies to $\delta a_{\mathrm{eq,dry}}/a_0 = -3(p-p_a)\,a_0/k - 2\alpha\gamma/k$, which is a result used in the main text.

Supplementary Text 2

Biological Pore Data

MscL

Negative compressibility data for MscL biological channel, reported in Table 1 of the main manuscript, are inferred from two different studies. The intrusion pressure p_{int} is inferred from experimental high hydrostatic pressure (HHP) patch-clamp recording by Petrov et al. [S1]; molecular insights of the open and closed pore structure are taken from the Molecular Dynamics analysis by Anishkin et al. [S2]. For reader convenience, we report here a combined figure with the main results from the two manuscripts, Fig. 5.

We considered the intrusion pressure as the points where the fitted linear open pore probability (black dashed line) exceeds 0.5, i.e. $p_{\mathrm{int}} = 40$ MPa, see Fig. 5A (red dotted lines). Extrusion pressure data are not available, since the hysteresis loop under high hydrostatic pressure were not characterized in the cited studies. The closed radius, a_0, and the expansion due to the opening of the pore, $\Delta a_{\mathrm{NC}}^{\mathrm{int/ext}}$, are obtained from the projected area A of the protein reported in panel c as $a_0 = \sqrt{A_{\mathrm{closed}}/\pi} = 25.24\,\text{Å}$ and $\Delta a_{\mathrm{NC}}^{\mathrm{int}} = a_1 - a_0$, with $a_1 = \sqrt{A_{\mathrm{open}}/\pi} = 35.24\,\text{Å}$ radius of the open pore.

Compressibility of proteins is estimated to be around $\beta = 180\,\mathrm{TPa}^{-1}$ [S3]. Considering $\frac{\Delta V}{V} = -\beta \Delta p$ with $V = \frac{4}{3}\pi a^3$, we can estimate a relative compression of the closed structure, from 1 atm to (before) the intrusion point, $\Delta a_{\mathrm{p}}^{\mathrm{int}}/a_0$, with $\Delta a_{\mathrm{p}}^{\mathrm{int}} = a_{\mathrm{p}} - a_0$ and $a_{\mathrm{p}} = a_0(1 - \beta\Delta p)^{1/3}$ compressed (gyration) radius, obtaining a value of about $\Delta a_{\mathrm{p}}^{\mathrm{int}}/a_0[\%] = |1 - (1 - \beta \Delta p)^{1/3}| \times 100 = 0.2\%$ at 40MPa.

BK

Negative compressibility data for BK biological channel, reported in Table 1 of the main manuscript, are inferred from the experiments of Macdonald[S4] and the molecular structures taken from Tao and MacKinnon [S5]. For reader convenience, we report here a combined figure with the main results from the two manuscripts, Fig. 6.

As for MscL, we considered the intrusion pressure as the points where the open pore probability exceeds 0.5. Open pore probability in Fig. 6 are reported as $\log_{10}(P_{\mathrm{o}}^*/P_{\mathrm{o}})$, with P_{o}^* open pore probability at high hydrostatic pressure and P_{o} the open pore probability at atmospheric pressure. Here, we consider $P_{\mathrm{o}} \simeq 1.5\%$, considering the intercept of the fitted curve (black line) in Fig. 6. Hence, the 50% open pore probability corresponds to $\log_{10}(50/1.5) = 1.5$, i.e. $p^{\mathrm{int}} = 1000$ atm $= 100$ MPa, see Fig. 6A (red star). Extrusion pressure data are not available, since hysteresis loop under high hydrostatic pressure where not characterized in the cited studies.

The closed radius, a_0, and the expansion due to the opening of the pore, $\Delta a_{\mathrm{NC}}^{\mathrm{int/ext}}$, are obtained from the gyration radius of the transmembrane region of the molecular structure reported in panel b as $\Delta a_{\mathrm{NC}}^{\mathrm{int}} = a_1 - a_0$, with $a_1 = R_{\mathrm{g}}^{\mathrm{open}} = 46$ Å and $a_0 = R_{\mathrm{g}}^{\mathrm{closed}} = 39.4$ Å.

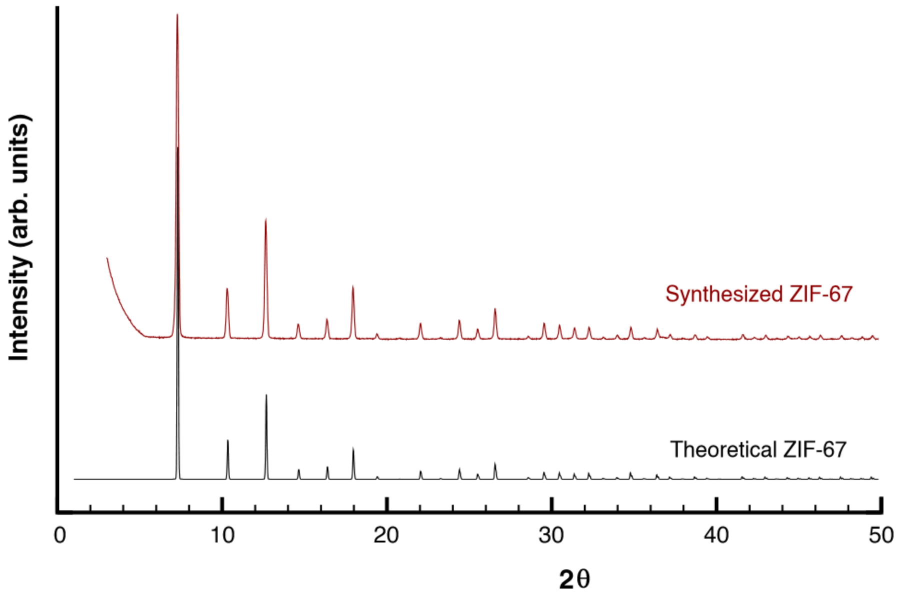

Supplementary Figure 1 Experimental XRD pattern of the synthesised ZIF-67 sample. Source data are provided as a Source Data file.

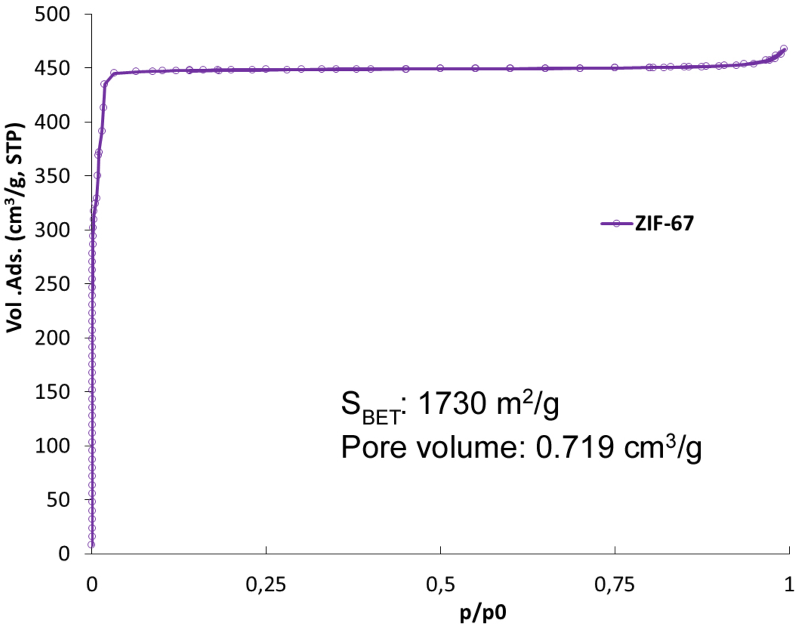

Supplementary Figure 2 N_2 adsorption experiment in ZIF-67. Source data are provided as a Source Data file.

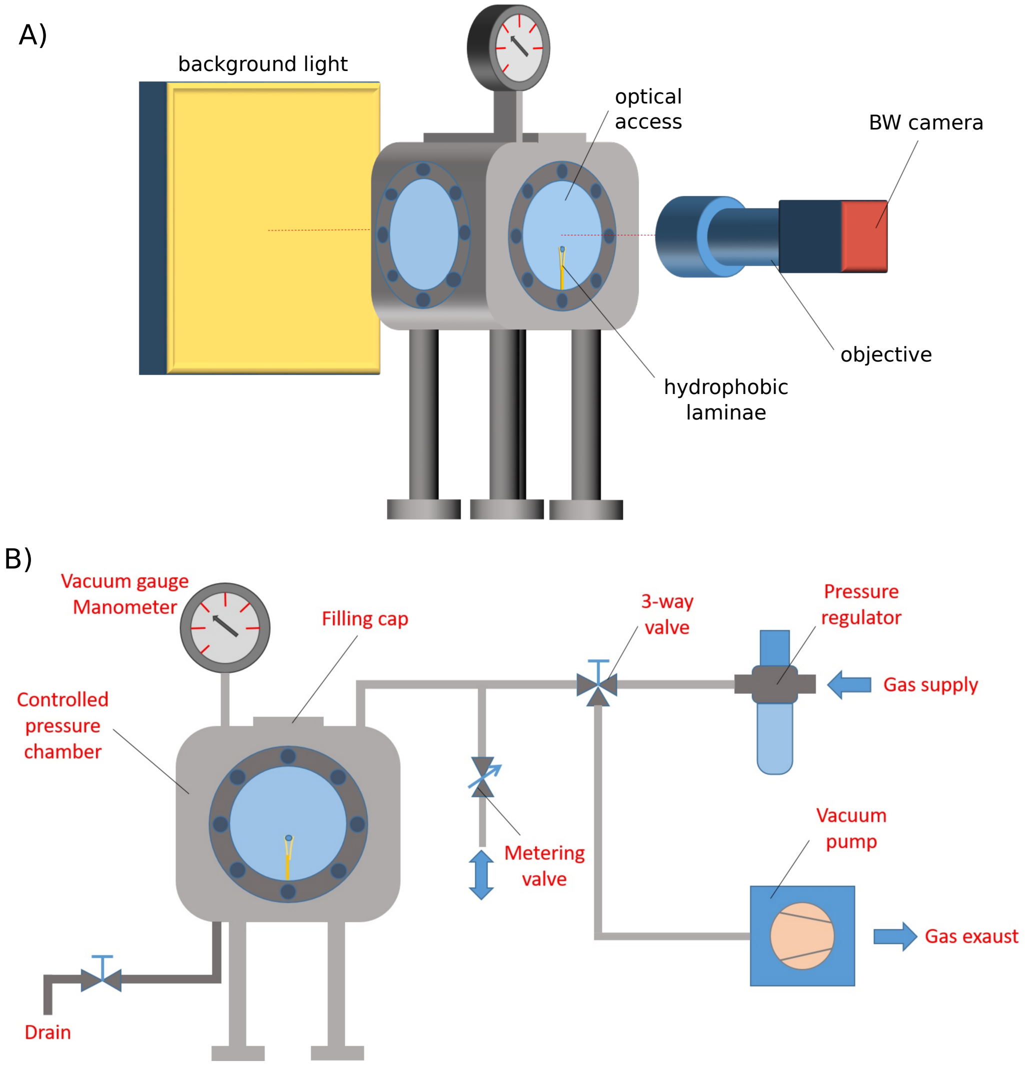

Supplementary Figure 3 Experimental setup used to characterise compression and decompression of the milliMES. (**A**) Camera setup; (**B**) detail of the compression/decompression circuit in the pressure cell. Pictures are realized by the authors with Microsoft PowerPoint.

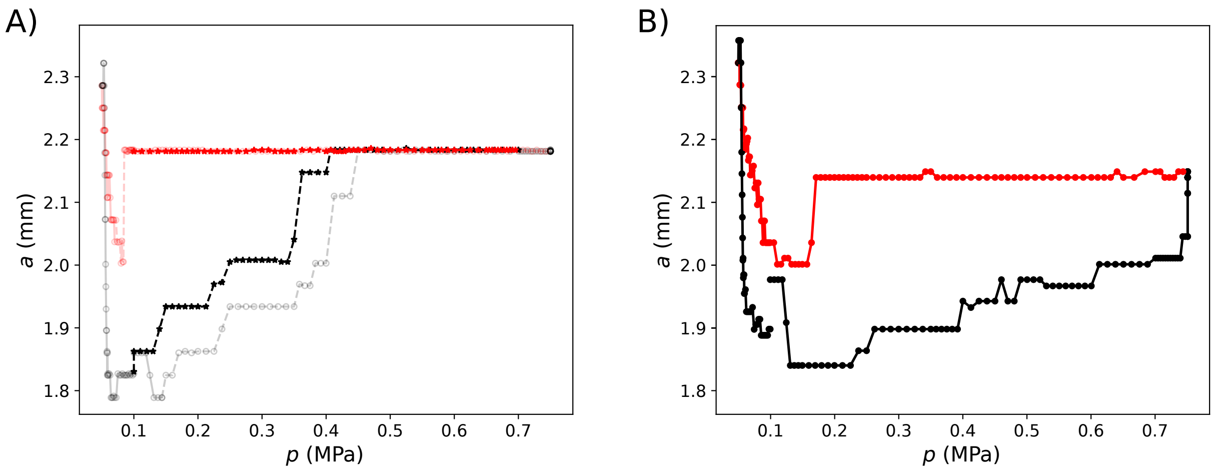

Supplementary Figure 4 Reversibility of intrusion and extrusion cycles of the milliMES. In black the compression stage, in red the decompression. (**A**) Same laminae used in the Fig. 4 of the main manuscript. The first cycle is reported in transparency, while the second is marked by stars. (**B**) Independent replica, different laminae of the same dimension. Source data are provided as a Source Data file.

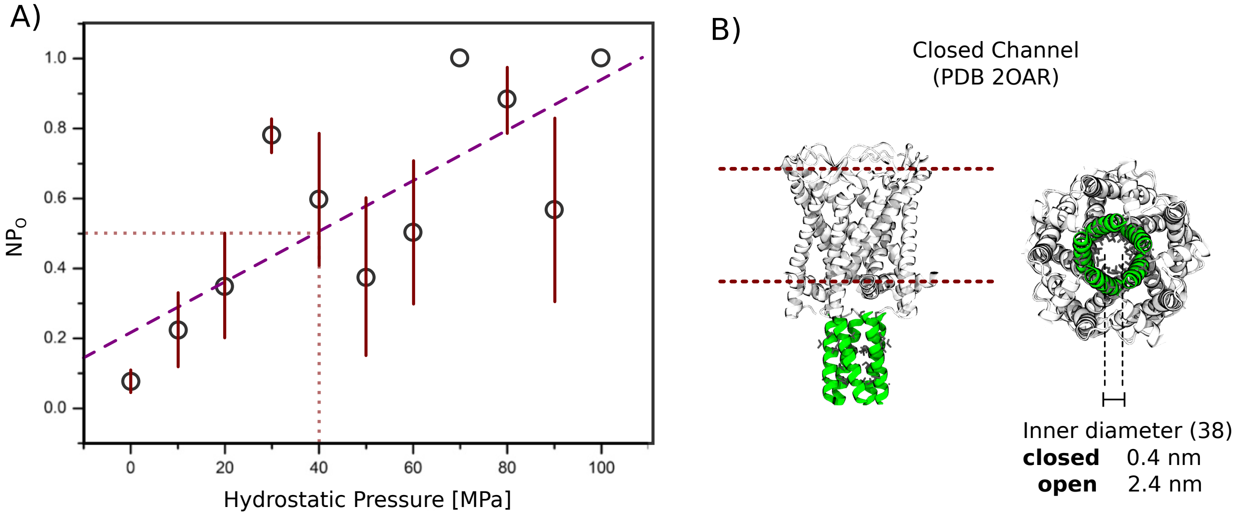

Supplementary Figure 5 MscL data from Petrov et al. [S1]. (**A**) Experimental open pore probability measured at high hydrostatic pressure, using a development of Heinemann's flying-patch patch-clamp technique. Data are measured from the spontaneously active gain-of-function (GOF) G22E mutant of MscL in situ in vitro by reconstitution into azolectin liposomes. For the Wild Type pore, it was reported that it needs much larger pressures to be opened [S1], but data were not shown in their study. (**C**) Molecular structures of the MscL channel in closed conformations (PDB 2oar [https://doi.org/10.2210/pdb2OAR/pdb]); render realised with VMD software.

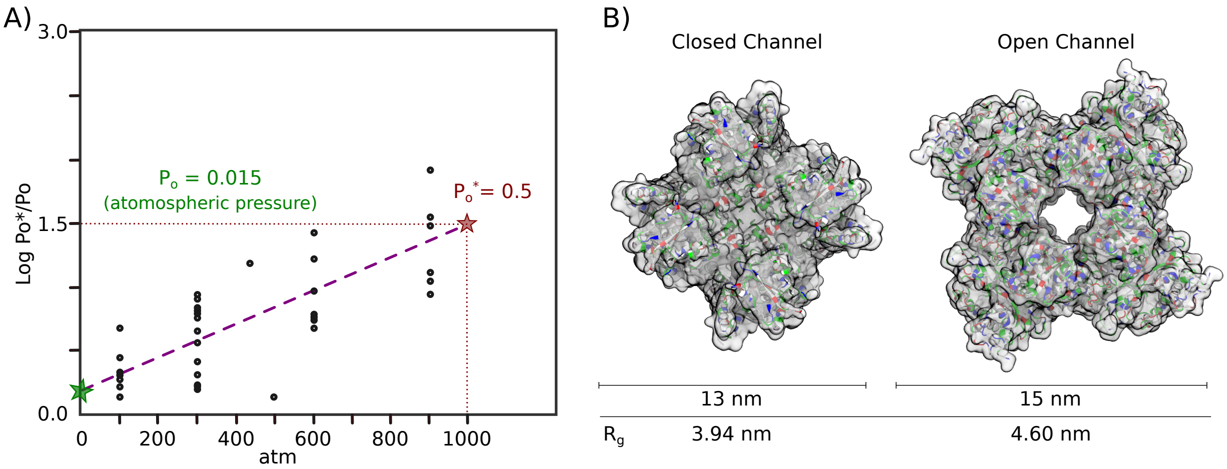

Supplementary Figure 6 BK data from Macdonald [S4] and from Tao and MacKinnon [S5]. (**A**) Open pore probability at high hydrostatic pressure for BK channel, adapted from Macdonald [S4]. Recording are conducted using a development of Heinemann's flying-patch patch-clamp technique. The red star represent the point where the fitted open pore probability, P_o^*, reaches 50%; the value is inferred considering an open probability at atmospheric pressure $P_o = 1.5\%$. (**B**) Molecular structures of the BK channel in closed and open conformations [S5], taken from the Protein Data Bank (PDB: 6V22 [https://doi.org/10.2210/pdb6V22/pdb], 6V35 [https://doi.org/10.2210/pdb6V35/pdb]). R_g is the gyration radius computed over the transmembrane region of the pore. Scale bars for the two systems are reported as reference lengths. Gyration radius and figure rendering are computed with VMD software.

Supplementary Movies

Supplementary Movie 1

Illustration of the negative compressibility concept during hydrostatic compression/decompression cycles.

Supplementary Movie 2

Video of the milliMES of Fig. 4 during the two compression/decompression cycles shown in Fig. S4A.

References

[S1] Petrov, E., Rohde, P. R. & Martinac, B. Flying-patch patch-clamp study of G22E-MscL mutant under high hydrostatic pressure. *Biophysical journal* **100**, 1635–1641 (2011).

[S2] Anishkin, A., Sukharev, S., Vanegas, J. M. *et al.* Mechanical activation of mscl revealed by a locally distributed tension molecular dynamics approach. *Biophysical journal* **120**, 232–242 (2021).

[S3] Kitchen, D. B., Reed, L. H. & Levy, R. M. Molecular dynamics simulation of solvated protein at high pressure. *Biochemistry* **31**, 10083–10093 (1992).

[S4] Macdonald, A. Effect of high hydrostatic pressure on the bk channel in bovine chromaffin cells. *Biophysical journal* **73**, 1866–1873 (1997).

[S5] Tao, X. & MacKinnon, R. Molecular structures of the human slo1 k+ channel in complex with β4. *Elife* **8** (2019).
