# Supplementary material for: Bubbles enable volumetric negative compressibility in metastable elastocapillary systems
Source: Nat Commun. 2024 Jun 13;15:5076. doi: 10.1038/s41467-024-49136-w (PMC11176325; doi:10.1038/s41467-024-49136-w)
Supplement: Supplementary file 4 — Description of Additional Supplementary Files [file 41467_2024_49136_MOESM4_ESM.pdf]

Description of Additional Supplementary Files

File Name: Supplementary Movie 1

Description: Illustration of the negative compressibility concept during hydrostatic compression/decompression cycles.

File Name: Supplementary Movie 2

Description: Video of the milliMES of Fig. 4 during the two compression/decompression cycles shown in Supplementary Fig. 4A.
