# Supplementary figures and images for: Bubbles enable volumetric negative compressibility in metastable elastocapillary systems
Source: Nat Commun. 2024 Jun 13;15:5076. doi: 10.1038/s41467-024-49136-w (PMC11176325; doi:10.1038/s41467-024-49136-w)

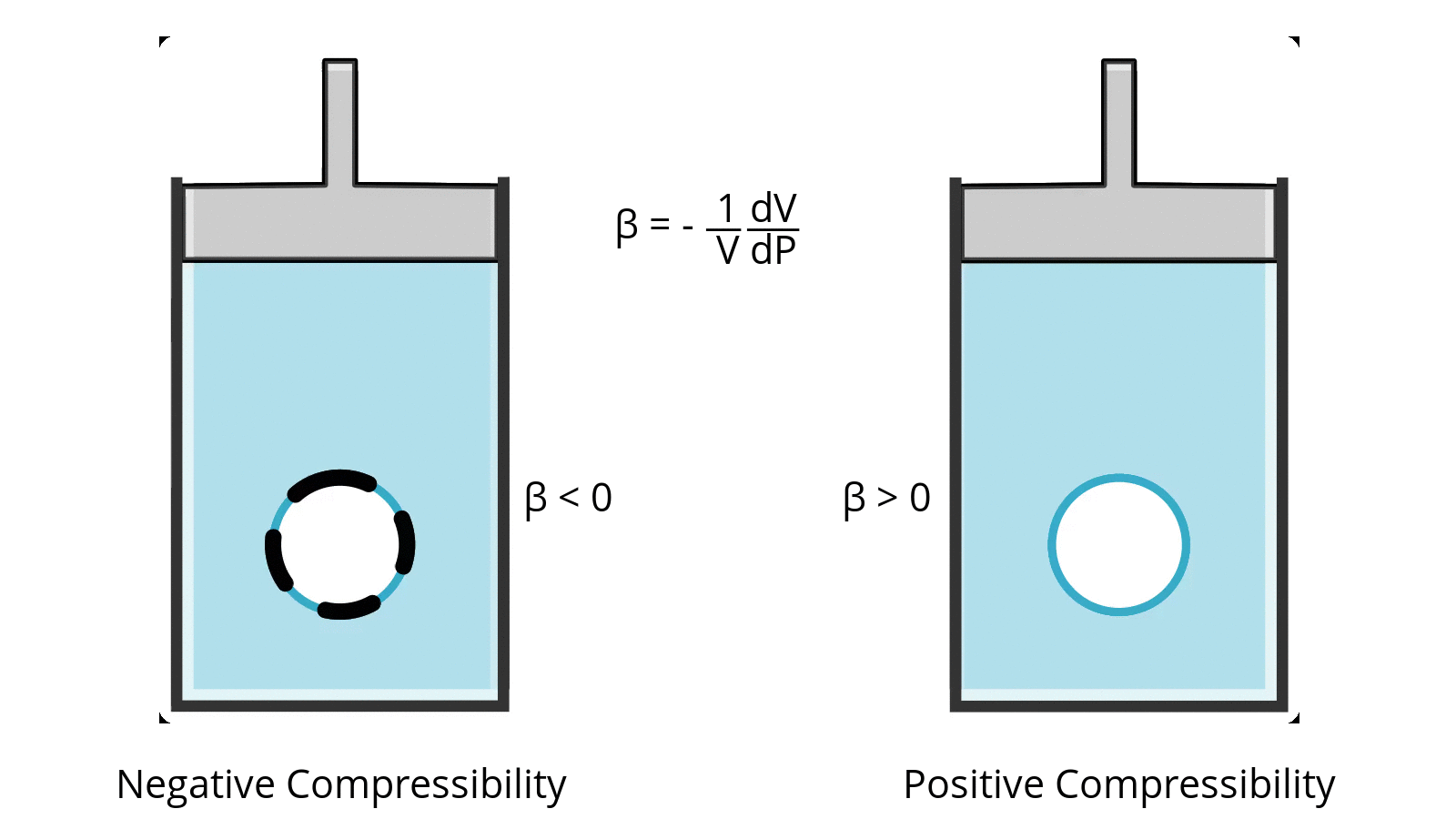

Supplement: Supplementary file 5 — Supplementary Movie 1 [file 41467_2024_49136_MOESM5_ESM.gif]
